# One-legged stance sway of older adults with and without falls

**DOI:** 10.1371/journal.pone.0203887

**Published:** 2018-09-17

**Authors:** Marcio R. Oliveira, Edgar R. Vieira, André W. O. Gil, Karen B. P. Fernandes, Denilson C. Teixeira, Cesar F. Amorim, Rubens A. da Silva

**Affiliations:** 1 Laboratory of functional evaluation and human motor performance (LAFUP) – UNOPAR, Professional Master’s in Physical Exercise in Health Promotion, Londrina, Paraná, Brazil; 2 Doctoral and Master’s Program in Rehabilitation Sciences UEL/UNOPAR, Londrina, Paraná, Brazil; 3 Department of Physical Therapy, Florida International University, Miami, Florida, United States of America; 4 Department of Physical Therapy, Pitagoras Unopar, Londrina, Paraná, Brazil; 5 Department of Physical Education, Universidade Estadual de Londrina, Londrina, Brazil; 6 Physical Therapy Master’s Program, Universidade Cidade de São Paulo (UNICID), São Paulo, Brazil; 7 Département des Sciences de la Santé, Programme de physiothérapie de l’université McGill offert en extension à l’Université du Québec à Chicoutimi (UQAC), et Laboratoire de recherche BioNR, Saguenay, Québec, Canada; University of Minnesota, UNITED STATES

## Abstract

Postural instability is a common problem among older people, and it is associated with mobility impairments, activity limitation and fear of falling. The evaluation of postural control can contribute to the early detection of balance deficits and help health professionals to manage this problem to prevent falls in older adults. The aim of this study was to identify center of pressure cut-offs to differentiate between older adults with and without falls in the past 12 months. The participants were 170 older adults (mean age 67 years, 50 fallers and 120 non-fallers). Center of pressure area and sway velocity in the anterior-posterior and medio-lateral directions were assessed using a force platform during three 30s one-legged stance trials with eyes open. The mean across trials was used for analysis. The time-limit (how long the participant was able to stay in one-legged stance, up to 30s) was also assessed. Fallers had poorer postural control than non-fallers (effect size ≥ 0.52, *P* <0.05). The cut-offs identified were 10.3 cm^2^ for Center of pressure area, 2.9 cm/s for velocity in the anterior-posterior, and 3.4 cm/s for medio-lateral velocity. The force platform parameters obtained an area under the curve of 0.72, with sensitivity of 78% and specificity of 68%. There were no significant differences between non-fallers and fallers for time-limit variable (17 seconds vs. 18 seconds). Force platform parameters during one-legged stance were associated with history of falls in older adults. The cut-offs obtained acceptable area under curve, sensitivity and specificity, with center of pressure area presenting the best performance to differentiate between fallers and non-fallers.

## Introduction

Falls are the most common cause of disability among older people and a public health problem with great social impact worldwide [1]. The consequences of falls include fractures, increased dependency, institutionalization, reduced functionality, and even death [2]. Assessment of the risk of falls in older adults is complex due to its multifactorial nature [3]. Several studies suggest a relationship between poor postural control and increased risk of falls in older adults [4]. Center of pressure (CoP) sway measures, which it is often used to determine postural control impairments, can differentiate younger and older adults [5]. In fact, CoP sway measures are valid and reliable signs of balance impairment among older people, and in turn, help identify people with increased risk of falls [6].

Older people with greater postural instability present increased risk of falls [7,8]. Pajala et al. evaluated 434 community-dwelling women (aged 63–76 years) during six bipedal standing balance tasks and found that participants with higher CoP sway measures had a two to fourfold greater risk of falls compared to participants with lower CoP sway measures. In addition, an inability to complete a tandem stance task was a significant predictor of falls [6]. Muir et al. reported that CoP sway measures during bipedal stance can reach cut-offs rate around 71% of sensitivity and 74% of specificity across fallers and non-fallers [7].

Although older adult postural control studies were often performed in bipedal standing [7,9]. One-legged stance is apparently more challenging and is a position often assumed during the activities of daily living such as walking, turning, climbing stairs and dressing [10]. Unfortunately, there is limited data related to cut-offs rates for one-legged stance using CoP sway measures to differentiate people with and without falls and/or those at risk for falls. Therefore, the aim of this study was to compare one-legged stance CoP sway measures of fallers and non-fallers, and to identify the cut-offs to differentiate fallers and non-fallers.

## Materials and methods

### Participants

Participants (170 older people) were recruited from the community in the city of Londrina, PR, Brazil. Inclusion criteria were (1) age > 60 years; (2) no lower limbs fractures, pain or disorders; (3) no impaired cognitive status (Mini-Mental State Examination scores ≥ 23); (4) no use of medication that could significantly alter their balance (such as phychotropic drugs), and (5) no sensory or neurological disorders. The exclusion criterion was the inability to perform the tests proposed (e.g. not able to stay in one-legged stance for 5 seconds).

A total of 170 older people participated in this study, of these 138 were female (81%). The subjects were divided into non-fallers (n = 120, of these 103 were female (86%)) and fallers (n = 50, of these 35 were female (70%)) based on the self-reported history of falls during the previous 12 months. We defined a fall as unintentionally coming to rest on the ground, floor, or other lower level [11].

Based on a previous CoP study [12] between non-fallers (1.65±0.1 cm^2^) and fallers (1.96±0.4 cm^2^), the minimal sample size for a power of 0.95 to run unpaired t-tests at the 0.05 significance level was 48 subjects per group. The protocol and the consent form were approved by the Ethics Committee on Research Involving Human Subjects from the *Universidade Norte do Parana* (protocol #0070/09).

The physical activity level of the individuals was evaluated by the number of steps performed and measured by means of a pedometer (DIGI-WALKER model SW700, Yammax, Japan) for seven days, placing it to the right at the waist toward the midline of the knee and the handgrip strength data was obtained with a Jamar Hydraulic Hand Dynamometer (Model J00105, Sammons Preston Inc., Illinois, USA) as described by Mendes et al. [13].

### Balance assessment

The initial interview and all balance assessments were performed by the same trained evaluator. Balance was quantified using a force platform (BIOMEC 400, EMG system do Brasil, SP, Ltda). The participants performed one-legged stance on the preferred leg, barefoot with their arms at their sides parallel to their trunk and with eyes open. A mark on the force platform was used to standardize the position of the feet [14]. During testing, the participant looked at a target (14.5 cm height by 14.5 cm wide and 4 cm thick) placed on a wall at eye level 2 m away. All participants performed three 30 s trials, with 30 s rest intervals between each trial [5]. To prevent falls, an investigator stood close to the volunteers during all tasks.

The vertical ground reaction force data from the force platform (Z forces) were sampled at 100 Hz. All force signals were filtered with a 35 Hz low-pass second-order Butterworth filter. The signals from the force platform sensors were converted into CoP data using computerized stabilography using MATLAB routines (The Mathworks, Natick, MA). The following balance parameters were calculated:

1. The 95% confidence ellipse area of CoP (A-CoP in cm^2^) which represents an ellipse area that includes 95% of all CoP positions during a trial;
2. The mean CoP sway velocity (VEL in cm/s) in the anteroposterior (A/P) and mediolateral (M/L) directions;
3. The time-limit which was defined as the maximum time (up to 30 s) each subject was able to stay in one-legged stance without losing balance, i.e., when the lifted foot touched the force platform.

These balance parameters were calculated for the total duration of each trial for each subject and the mean of the three trials was analyzed. The psychometric properties of these measures are adequate [5,15].

### Statistical analysis

Shapiro-Wilk test was performed to analyze normality of the data distribution. Results were presented as median (interquartile range 25–75%) or absolute and relative frequency (%). Differences between groups were investigated using Mann Whitney test (CoP parameters in non-fallers x fallers) or Chi-square test as appropriate (gender x falls). Receiver operating characteristics (ROC) curves were used to determine cut-off values based on CoP parameters related to sensitivity and specificity to identify the participants as fallers or non-fallers. The area under the curve (AUC) describes the test’s overall performance. Hosmer and Lemeshow [16] suggested that an AUC ≥ 0.7 indicates acceptable discriminatory power, and an AUC of 0.50 corresponds to random classification. The input data in the statistical program were 0 non-fallers and 1 faller (value of state variable). The effect size (*d*) between groups was calculated to determine the magnitude of effects using the equation: *d* = m_1_ –m_2_ / SDm_2_, where m_1_ is the mean of the fallers group, while m_2_ is the mean of the non-faller group, and SDm_2_ is the standard deviation of the non-faller group. Statistical analyses were carried out using SPSS v.21 (SPSS, Inc., Chicago, IL, USA) and MedCalc 17.1, (MedCalc Software, Ostend, Belgium) and significance was set at *P* <0.05.

## Results

There were no significant differences between the groups (non-fallers and fallers) for age, weight, height, Body Mass Index (BMI), and mini-mental scores variables (Table 1).

**Table 1:** Characteristic of the participants.

Variable	All subjects (n = 170)	Non-fallers (n = 120)	Fallers (n = 50)	*P* value
Age (years)	67[63–72]	67[63–72]	68[64–74]	0.472
Weight (kg)	68[58–77]	67[57–76]	66[58–77]	0.960
Height (m)	1.56[1.50–1.63]	1.55[1.50–1.60]	1.58[1.50–1.65]	0.072
BMI (kg/m^2^)	27[24–31]	27[24–30]	26[24–29]	0.145
Mini mental	24[22–27]	24[22–26]	24[22–27]	0.755

The data are presented in median and interval interquartile range [25–75]. BMI: Body Mass Index.

The findings did not change when BMI was included as a covariate (F: 0.51–1.28, P = 0.258–0.822). The prevalence of falls was 29%, with 120 non-fallers and 50 fallers. In general, the participants were considered as low active (men = 7.044 and women = 7.215 steps; low active = 5.000–7.499 steps/day)[17] and for handgrip no difference was found between men and women (median 25 Kgf, 95% CI 24–31 and median 25 Kgf, 95% CI 25–28, P = 0.862; respectively). Significant differences (P <0.03, Mann Whitney test) were found between sexes for all postural control measures. Women presented better postural control than men.

**Fig 1:**
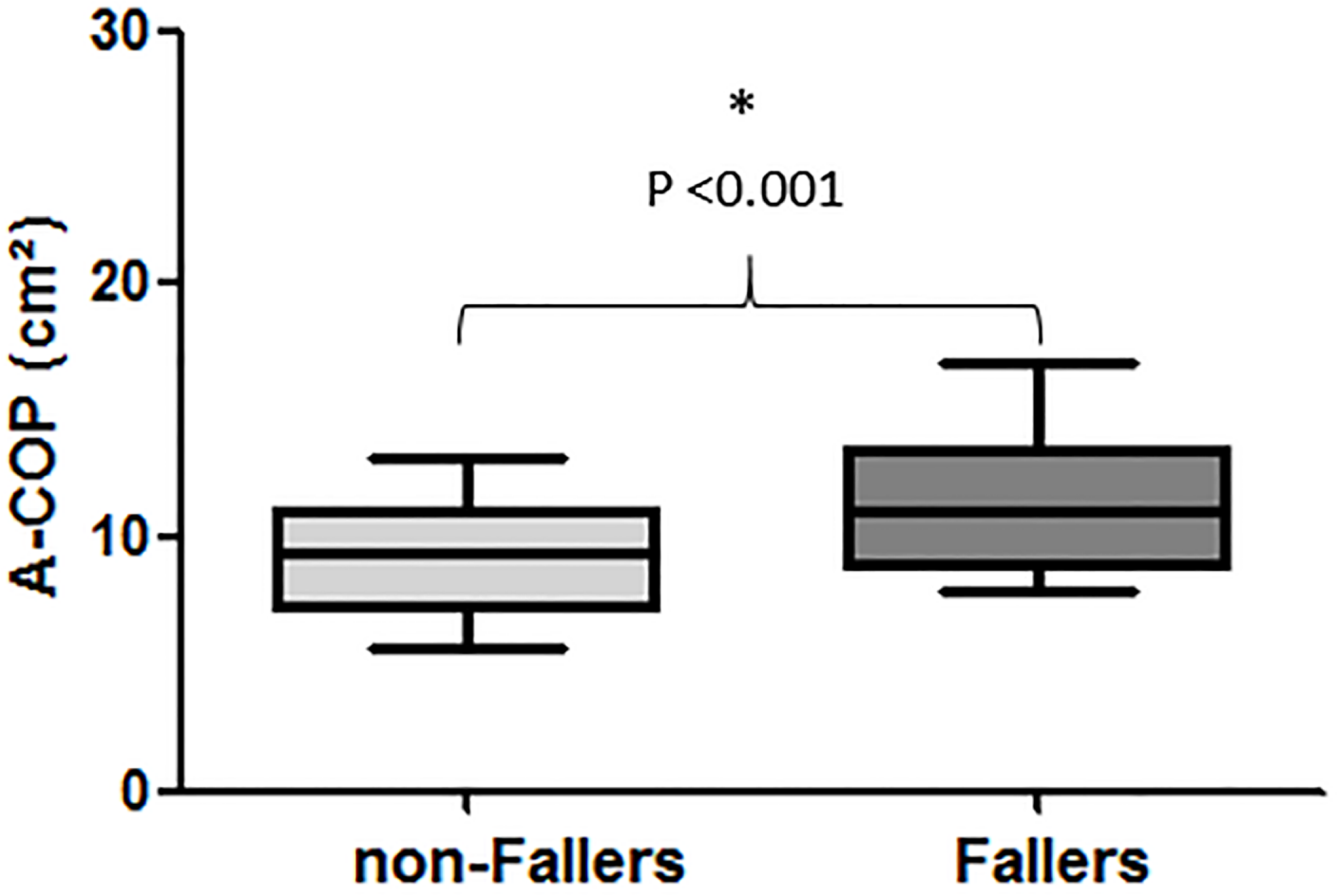
Comparison between groups (non-fallers and fallers) for the area of center of pressure (A-COP) variable.

**Fig 2:**
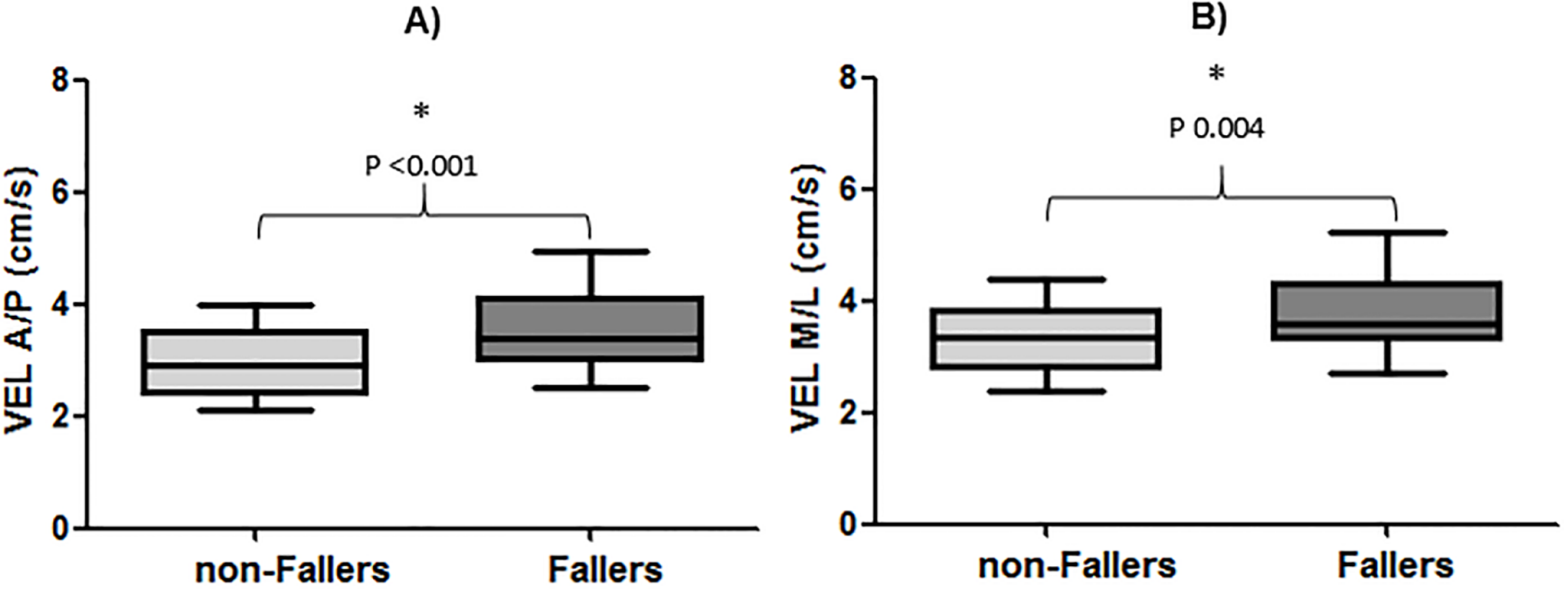
Comparison between groups (non-fallers and fallers) for the variables: A) Center of pressure velocity in the anteroposterior (VEL A/P) and B) mediolateral (VEL M/L) directions.

Significant differences (*P* <0.05) between groups were found for all CoP parameters; fallers obtained higher CoP values (poorer balance) than non-fallers (A-CoP, *d* = 0.93; VEL A/P, *d* = 0.71; VEL M/L, *d* = 0.52; see Figs 1 and 2. However, there were no significant differences (*P* = 0.749) between groups for time-limit (non-fallers = 18 s [11–24] vs. fallers = 17 s [11–20], *d* = -0.10).

Table 2 shows the results for the area under the ROC curve (AUC), including the sensitivity and specificity, for the CoP parameters. The AUC varied from 0.65 to 0.72, with the sensitivity reaching 78% for VEL A/P, while the specificity of 68% for the A-CoP. The cut-off points for the CoP parameters were >10.30 cm^2^ for A-CoP, >2.93 cm/s for VEL A/P, and >3.40 cm/s for VEL M/L.

**Table 2:** Area under curve, sensitivity, specificity for the force platform variables.

Force plate variable	AUC	Sensitivity (%)	Specificity (%)	95% CI	P	cut-off
A-COP (cm^2^)	0.72	66	68	0.64–0.78	< 0.001*	> 10.3
VEL A/P (cm/s)	0.68	78	54	0.60–0.76	0.001*	> 2.9
VEL M/L (cm/s)	0.65	70	58	0.57–0.72	0.002*	> 3.4

Receiver Operating Characteristic (ROC) curve analysis. AUC: Area Under the Curve; 95% CI: confidence interval; Area of center of pressure (A-COP); COP velocity in the anteroposterior (VEL A/P) and mediolateral (VEL M/L) directions.

*Statistical significant differences between those with and without falls (n = 170).

Table 3 shows the results for the area under the AUC individually for each sex. The A-CoP was again the variable with better discriminatory value for both women and men (AUC: 70 and 75 respectively), with sensitivity reaching 94% and specificity 60% for women, while that 100% and 76%, respectively for men. When separated for sex, the cut-off points were: for women >7.8 cm^2^ for A-CoP, >2.9 cm/s for VEL A/P and >3.4 cm/s for VEL M/L; while for men >10.8 for A-CoP, >2.9 cm/s for VEL A/P and >3.2 cm/s for VEL M/L. However, only A-CoP had an acceptable discriminatory power (AUC ≥0.7) between fallers and non-fallers.

**Table 3:** Area under curve, sensitivity, specificity for the force platform variables separated for sex (n = 32 male and 138 female).

Force plate variable		AUC	Sensitivity (%)	Specificity (%)	95% CI	P	cut-off
A-COP (cm^2^)	M	0.75	73	76	0.56–0.88	0.006	> 10.8
	F	0.70	94	38	0.62–0.78	< 0.001	> 7.8
VEL A/P (cm/s)	M	0.68	97	35	0.49–0.83	0.060	> 2.9
	F	0.64	69	57	0.55–0.71	0.010	> 2.9
VEL M/L (cm/s)	M	0.69	100	41	0.50–0.84	0.033	> 3.2
	F	0.58	57	60	0.49–0.66	0.171	> 3.4

Receiver Operating Characteristic (ROC) curve analysis. AUC: Area Under the Curve; 95% CI: confidence interval; Area of center of pressure (A-COP); COP velocity in the anteroposterior (VEL A/P) and mediolateral (VEL M/L) directions; M: male and F: Female.

## Discussion

This study was conducted to compare one-legged stance CoP sway measures of fallers and non-fallers, and to identify the cut-offs to differentiate fallers and non-fallers. Fallers had poorer balance than non-fallers. The CoP parameters were more sensitive than time-limit, but only A-CoP had sufficient accuracy (AUC ≥ 0.7) to differentiate the groups (fallers and on-fallers) in both men and women.

The prevalence of falls in the study was 29% with a great proportion for men than women (47% vs 25% respectively). In general, fall events occur more in women than men and is associated with the worst comorbidity values such as hypovitaminosis, weakness, mental state, medication, problems with hearing or vision, fat body mass [18–20]. Pereira et al. demonstrated that when similar conditions of health and physical fitness are considered, the men were more than twice as prone to fall as the women [19]. This may help explain our results because the characteristics of the subjects of the present study were similar (P> 0.05), although some comorbidities were not evaluated in this study. On the other hand, BMI variable did not affect older adults’ one-legged stance postural control. We did not measure waist circumference and this variable may be target of investigation in future research. Furthermore and consistently with previous findings, men presented significantly poorer balance than women [21].

The time-limit variable computed in parallel to CoP data in the one-legged stance was not sensitive to differences in balance between non-fallers and fallers for this balance assessment condition. Our findings were in agreement with some previous studies [22], but contrary to other studies [23]. Hurvitz et al. showed that older adults with a history of falling had a time-limit of 9.6 s while those without a history of falling had a time-limit of 31 s [23]. The discrepancies between our findings and those of Hurvitz et al. are related to a pathological presence in their sample (ex: subjects with peripheric neuropathy), sex factor (89% of their sample were men while in the present study 80% were women), and age concerns (in their study the fallers were 70 yrs old and non-fallers 63 yrs old; *P* < 0.01; while in the present study no difference in age was reported between groups). These results suggest that quantified measures of balance parameters, beyond time, are more sensitive to identify postural control impairments in older people. [24]. In addition, a recent study also reported the sensitivity of a variety of CoP variables (ex: CoP path length, CoP velocity sway) to distinguish older adult fallers versus non-fallers [25]. CoP parameters from a force platform can directly analyze balance deficits related to proprioception and postural adjustments (feedback and feedforward) of the neuromuscular system [10], which is limited for functional balance tests as a time-limit score, as previously supported by Nguyen et al. [26].

Only one force platform variable (A-CoP) showed results with AUC >70 in this study. The discussion between the variables analyzed (A-CoP and VEL A/P, M/L) for AUC is limited because no study (our knowledge) had analyzed this topic during one-legged stance. Laessoe et al. found a limited cut-off score from a battery of tests for elderly including standing balance (time-limit), stepping ability, dual task, gait variability and others [27]. The authors reported a sensitivity of only 50%, while the specificity did not exceed 43% of the cases (values lower than those found in our study). Audifren et al. using an algorithm model (advanced probability and machine learning theory) from a five dimensional description of the statokinesigram on a wii balance board, reported an AUC of 0.75 for classifying fallers and non-fallers. In this case, the AUC was higher than our study (for all group (AUC 0.72) but similar with result found in men (AUC 0.75), however, the equipment (wii balance board), age of individuals (in mean 83 yrs) and balance condition (two legged stance) are different of the presented in this study [28]. It is important to emphasize that when the authors above used indices alone, the results of the ROC analysis were not good (AUC between 0.49–0.54). In fact, only a few studies reported positive results for predicting falls with these parameters but without the use of a challenge balance task as one-legged stance [6].

Compared to results of the present study (sensitivity of 78% and specificity of 68% across CoP parameters), Muir et al. reported a sensitivity of 71% and specificity of 74% from their CoP values for identifying those with an increased risk of falls [7]. However, the authors reported these results for a two-legged standing condition. In fact, the strengths of the present study over past works are based in acceptable results with CoP parameters during a one-legged stance condition, which represents at least part of daily living activities such as step walking and dressing and more challenging for the postural control system.

Howcroft et al. reported cut-off results (ROC curve) of 100 older subjects (76 yrs), but did not specify whether those elderly individuals who presented values above the cut-off point fell after six months of follow-up [9]. The importance of this study is to guide new studies (prospective) to identify if older people that showed values above of cut-off points had falls when evaluated in one legged stance on force platform. The early detection of elderly adults with potential for falling may help allow timely referral to an appropriate fall prevention program [29].

Finally, our study presents some limitations. Our sample was characterized by a higher percentage of women, so more studies including a number elevated of men or paired to women would be necessary. Only one challenge balance condition was tested. Other challenge tasks such as trunk perturbation and dynamic postures should be also considered in the future studies for comparison with one-legged stance. In general, our results cannot necessarily be generalized to all older adults because: 1) Our sample (n = 50) does not represent the entire heterogeneity of balance status in the population context of older individuals, and 2) Older adults with neuro-musculoskeletal disorders were not evaluated. Our falls data are also based on a retrospective analysis of falls in a self-reporting context and the location about falls (at home or outside of the home) no was explored.

## Conclusion

Our findings showed that fallers have poorer postural control compared to non-fallers during a one-legged stance task in the CoP parameters. The cut-offs had acceptable AUC, sensitivity and specificity, with A-CoP presenting the best performance to differentiate between fallers and non-fallers. These findings can be considered preliminary in the area of balance deficits in older adults (apparently healthy) until prospective studies can further validate our results.

## Supporting information

S1 Fig Receiver operating curve to area of center of pressure (A-COP) variable. (TIF)

S2 Fig Receiver operating curve to center of pressure velocity in the anteroposterior (VEL A/P) direction. (TIF)

S3 Fig Receiver operating curve to center of pressure velocity in the mediolateral (VEL M/L) direction. (TIF)
