# Supplementary figures and images for: One-legged stance sway of older adults with and without falls
Source: PLoS One. 2018 Sep 17;13(9):e0203887. doi: 10.1371/journal.pone.0203887 (PMC6141084; doi:10.1371/journal.pone.0203887)

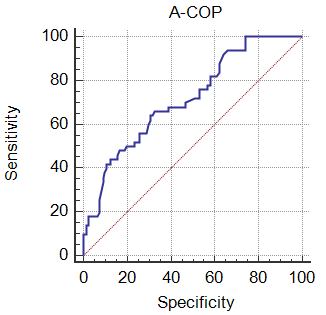

Supplement: S1 Fig — (TIF) [file pone.0203887.s001.tif]

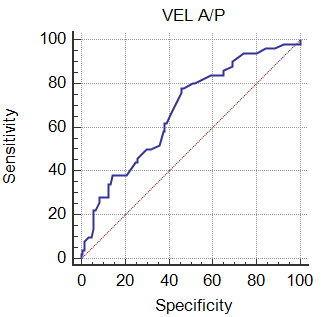

Supplement: S2 Fig — (TIF) [file pone.0203887.s002.tif]

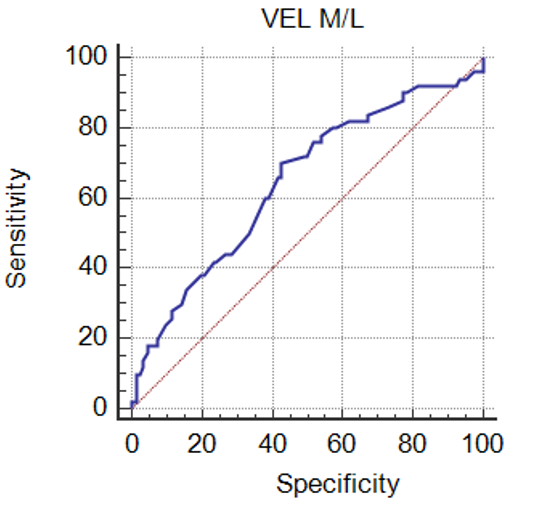

Supplement: S3 Fig — (TIF) [file pone.0203887.s003.tif]
